# Relationship Between Initial Urine Output and Mortality in Patients Hospitalized in Cardiovascular Intensive Care Units: More Is Not Better

**DOI:** 10.3389/fcvm.2022.853217

**Published:** 2022-04-25

**Authors:** Le Li, Zhenhao Zhang, Yulong Xiong, Zhao Hu, Shangyu Liu, Bin Tu, Yan Yao

**Affiliations:** National Center for Cardiovascular Diseases, Fu Wai Hospital, Peking Union Medical College, Chinese Academy of Medical Sciences, Beijing, China

**Keywords:** urine output, mortality, cardiovascular critical care, LOWESS smoothing, logistic regression

## Abstract

**Backgrounds:**

Decreased urine output (UO) is associated with adverse outcomes in certain patients, but this effect in patients admitted for cardiovascular diseases is still unproven. Moreover, the relationship between increased UO and prognosis is also unclear.

**Objective:**

To investigate the relationship between decreased or increased UO and outcomes in patients with the cardiovascular intensive care unit (CICU).

**Methods:**

This study was a retrospective cohort analysis based on the medical information mart for intensive care III (MIMIC-III) database. The patients' data were extracted from the Beth Israel Deaconess Medical Center (Boston, MA) between 2001 and 2012. With the initial 24-h UO range from 0.5 to 1.0 ml/kg/h as the reference, participants were divided into the several groups. The primary outcome was 30-day mortality. The secondary outcomes were 90-day mortality, ICU mortality, hospital mortality, use of mechanical ventilation (MV), and vasopressor agents in the first 24-h of ICU. The association between UO and mortality was assessed by multivariable logistic regression.

**Results:**

A total of 13,279 patients admitted to CICU were included. Low UO (< 0.5 ml/kg/h) was strongly associated with 30-day mortality (unadjusted OR = 3.993, 95% CI: 3.447–4.625, *p* < 0.001), and very high UO (≥ 2.0 ml/kg/h) was also a significantly risk factor for 30-day mortality (Unadjusted OR = 2.069, 95% CI: 1.701–2.516, *p* < 0.001) compared with the reference. The same effects also were shown in the multivariable logistic regression, adjusted by age, gender, vital signs, common comorbidities, and use of diuretics, with an adjusted OR of 2.023 (95% CI: 1.693–2.417, *p* < 0.001) for low UO and 1.771 (95% CI: 1.389–2.256, *p* < 0.001) for very high UO. Moreover, both decreased UO and increased UO were risk factors for 90-day mortality, ICU mortality, hospital mortality, use of MV and vasopressor agents.

**Conclusion:**

The decreased and increased UO both were significantly associated with short-term mortality, the relationship between UO and mortality was U-shape rather than linear.

## Introduction

The 24-h urine output (UO) is an effective biomarker to judge the condition of patients hospitalized in the cardiovascular intensive care unit (CICU), especially for the patients with heart failure (HF) or acute myocardial infarction (AMI). Increased UO and reduced fluid intake (FI) would relieve cardiac load and then alleviate clinical symptoms of the patients significantly and fleetly (1–3). In addition, decreased UO is an important predictive indicator for acute kidney disease (AKI) and is highly associated with the poor prognosis of the patients admitted to CICU (4).

The majority of CICU patients are accompanied by the cardiac systolic or diastolic dysfunction, with reduced effective circulatory volume (ECV). Kidneys are vulnerable to ischemia, reduced ECV could activate renin-angiotensin-aldosterone system (RAAS) and then increase sodium–water reabsorption and decrease UO (5). The compensation, however, will increase cardiac volume load and exacerbate cardiac dysfunction, which leads to further reduction in the renal perfusion. The complicated and bilateral regulation mechanism is defined as cardiorenal syndrome (6). And there are limited therapeutic regimens for cardiorenal syndrome, some of them have to receive renal replacement therapy, leading to adverse outcomes. Accordingly, much more attention should be paid to the UO of CICU patients.

The previous studies have demonstrated that decreased UO was a key risk factor for poor prognosis of patients hospitalized in ICU (7–9), which was independent of AKI and diuretic use (10). Nevertheless, the population of the aforementioned studies lacks specificity. As previously mentioned, CICU patients are prone to oliguria or even anuria, which in turn worsens cardiac dysfunction and clinical symptoms. Therefore, it is necessary to evaluate the relationship between UO and prognosis in patients admitted to CICU. The previous study suggested that low UO may be associated with adverse outcomes and increased UO could be a protective factor (10). However, overmuch UO could reduce ECV, leading to hypoperfusion of tissues and organs, hypotension and even shock, which may have adverse effects on prognosis (11). There is no study on the correlation between increased UO and prognosis in CICU patients. Therefore, we designed a cohort study to investigate the relationship between UO and short-term mortality in the patients admitted to CICU and to verify whether the relationship was linear.

## Materials and Methods

### Source of Data

Medical Information Mart for Intensive Care III (MIMICIII, version 1.4) is a large single-center database containing de-identified health-related data of more than 40,000 ICUs patients admitted to the Beth Israel Deaconess Medical Center (Boston, MA, USA) between 2001 and 2012. The MIMIC-III, a freely accessible critical care database, contains medical information including patient demographics, clinical measurements, laboratory tests, prescription data, survival data, etc. (12). All the data are collected automatically by computer in the course of daily clinical work, and medical staff are not involved in data collection. Researchers who completed and passed an online course on “Protecting Human Research Subjects” organized by the National Institutes of Health (NIH) are qualified for inquiring the information from this database. And the author (LL) obtained the qualification (record ID: 35965741) and was responsible for data extraction. The Massachusetts Institute of Technology approved the establishment of the database, therefore, the patient informed consent was not applicable.

### Study Population

We conducted a retrospective cohort study based on the MIMIC-III database, patients who were hospitalized in the CICU including coronary care unit (CCU) and the cardiac surgery recovery unit (CSRU) were included in this study. And patients aged < 18 years or without urine output data were excluded.

### Data Collection and Outcome Definition

Medical information was extracted from the MIMIC-III database by PostgreSQL tools version 13.0. There were some patients who had ICU admission records more than once, the first ICU admission was selected for analysis. The subject IDs were used to identify distinct patients. Patient demographics, vital signs, intervention, common causes for cardiac ICU admission, common comorbidities, laboratory tests, urine output, use of diuretics, and survival data were collected. The first records in ICU admission of the aforementioned data were selected for analysis. In this study, congestive heart failure (CHF) could be divided as acute/chronic/acute on chronic episodes and systolic/diastolic CHF; ventricular arrhythmia (VA) included ventricular tachycardia (sustained or paroxysmal) and ventricular flutter or fibrillation; acute kidney disease (AKI) was diagnosed based on the following clinical practice guidelines: increase in serum creatinine (SCr) by ≥0.3 mg/dl in 48 h, increase in SCr to 1.5 times over baseline levels in 7 days, and patient UO < 0.5 ml/kg/h for 6 h; chronic kidney disease (CKD) included stages 1–5; sepsis was diagnosed based on the current guideline (13); liver disease included hepatitis and hepatic cirrhosis; stroke included cerebral infarction and hemorrhage; and cancer included any end-stage malignancy. The mean of first 24-h UO <0.5 ml/kg/h was categorized as low UO, and UO between 0.5 and 1.0 ml/kg/h was set as the reference group. In order to determine the correlation between UO and prognosis in detail, UO which ≥1.0 ml/kg/h was divided into three groups (1.0–1.5 ml/kg/h, 1.5–2.0 ml/kg/h, ≥ 2.0 ml/kg/h). And UO ≥ 2.0 ml/kg/h was defined as very high UO.

The primary outcome was 30-day mortality. The secondary outcomes included 90-day mortality, ICU mortality, hospital mortality, use of mechanical ventilation (MV), and vasopressor agents in the first 24-h of ICU admission. And seven subgroup analyses were also performed based on the main causes and comorbidities for CICU admission.

### Statistical Analyses

The Kolmogorov–Smirnov test was used to evaluate the normal distribution of the data. Continuous variables are expressed as means ± SDs and compared using *t*-test. Levene's homogeneity of variance test was used to test the assumption of homoscedasticity. If the homoscedasticity was unsatisfied, the Welch's *t*-test was used for comparison between groups. Categorical data are expressed as proportions and were compared using the chi-squared test. A multivariable logistic regression (LR) was used for the covariate adjustment. The logistic models were built using the stepwise backward method. Variables with *P* < 0.05 in the univariate analyses were included in the multivariable analysis. And a stepwise backward elimination method was used to remove variables with *P* > 0.05. The Lowess Smoothing technique was used to explore the crude relationship between UO and mortality. The marginal effects of the UO to predict death probability were also conducted. In addition, the variance inflation factor (VIF) was assessed among the covariates in the logistic model, and VIF > 4.0 was interpreted as indicating multicollinearity. Variables with VIF > 4.0 were not included in the multivariate logistic regression. A two-tailed test was performed, and a *P* < 0.05 was considered to reflect statistical significance. All the statistical analyses were performed using Stata version 15.0 (StataCorp, College Station, TX, USA), R software version 4.0.4 (R Foundation for Statistical Computing, Vienna, Austria) and Python version 3.9 (Python Software Foundation, www.python.org).

The medical data from the MIMIC-III database had abnormal and missing values. The extreme and error values failing the logic check were censored and replaced with the mean values. The variables with the missing values accounting for more than 30% of the sample size were excluded. We used the mean imputation method to deal with missing values which was <5% of the sample size. We also built the random forest multiple interpolation (RFMICE) model using the Python software to fill the variables with missing values of 5–30%.

## Results

### Baseline Characteristics

**Figure 1:**
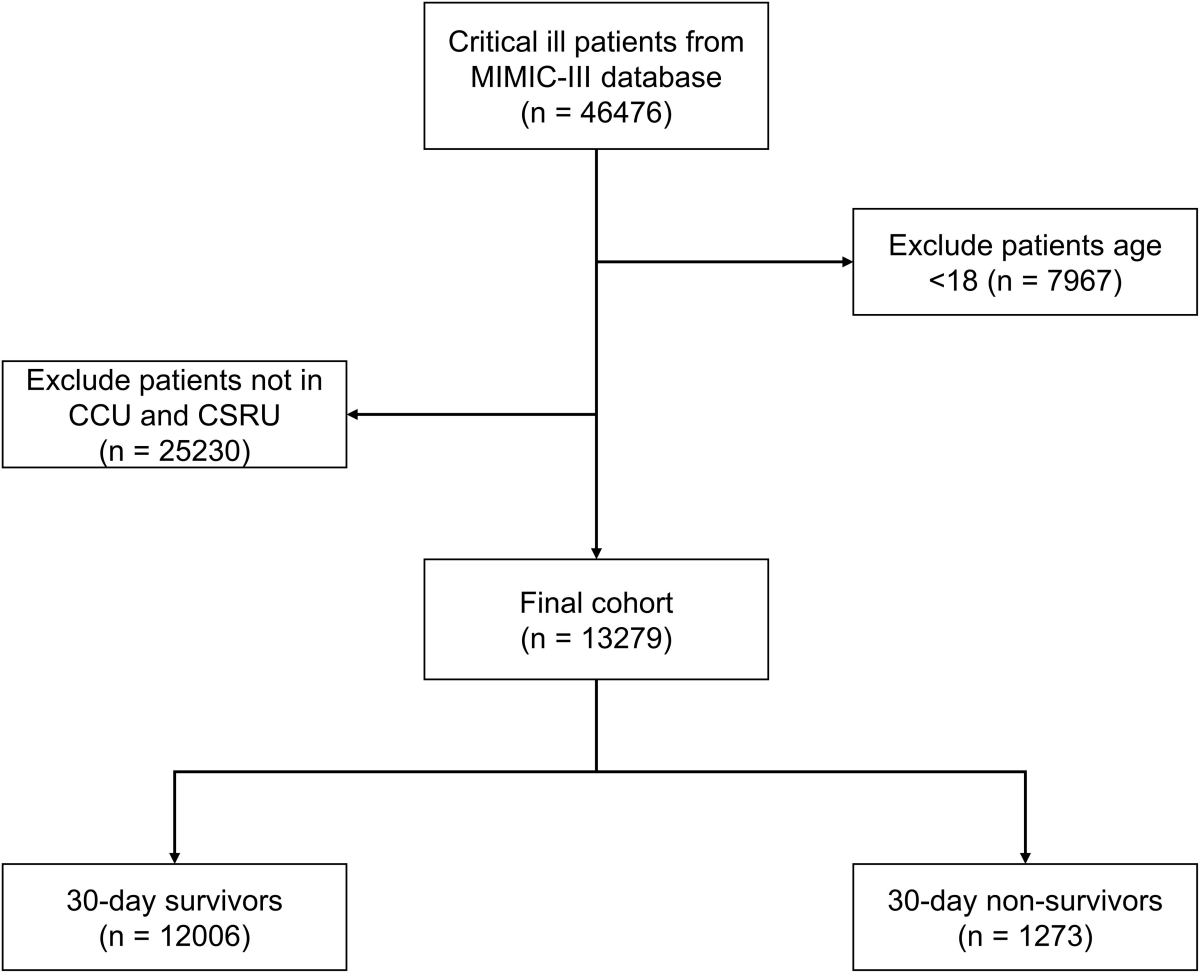
Flow chart of the study participants.

A total of 13,279 patients hospitalized for cardiovascular diseases were analyzed (Figure 1). There were 1,273 patients died within 30 days of discharge. Mean age of the all the participants was 67.3 ± 14.3 years old, and the non-survivor group was significantly higher than the survivor group (74.1 vs. 66.6, *p* < 0.001). The Simplified Acute Physiology Score (SAPS-II) and Sequential Organ Failure Assessment (SOFA) score in the dead group were also higher than the alive group (22.2 vs. 18.0 and 6.3 vs. 3.9, respectively, all *p* < 0.001). Moreover, UO in the non-survivor group was statistically lower than the survivor group (0.91 vs. 1.10 ml/kg/h, *p* < 0.001). Other comparisons of demographics and clinical characteristics between two groups were shown in Table 1.

**Table 1:** Comparisons of demographics between survivors and non-survivors.

**Variables**	**Total (*n* = 13,279)**	**Survivors (*n* = 12,006)**	**Non-Survivors (*n* = 1,273)**	***P*** **-Value**
**Demographics and characteristics**				
Age, years	67.3 ± 14.3	66.6 ± 14.2	74.1 ± 13.7	<0.001
Male, %	8,339 (62.8)	7,652 (63.7)	687 (54.0)	<0.001
Weight, kg	82.2 ± 19.6	82.7 ± 19.5	77.5 ± 20.7	<0.001
Caucasians, %	9,306 (70.1)	8,483 (70.7)	823 (64.7)	0.001
CCU, %	5,673 (42.7)	4,800 (40.0)	873 (68.6)	<0.001
CSRU, %	7,606 (56.1)	7,206 (60.0)	400 (31.4)	<0.001
SAPS	18.4 ± 5.2	18.0 ± 4.9	22.2 ± 6.1	<0.001
SOFA	4.2 ± 2.8	3.9 ± 2.6	6.3 ± 3.8	<0.001
**Interventions**				
Mechanical ventilation use (1st 24 h)	7,914 (59.6)	7,187 (59.9)	727 (57.1)	0.057
Vasopressor use (1st 24 h)	7,490 (56.4)	6,682 (55.7)	808 (63.5)	<0.001
**Vital signs**				
Temperature, °C	36.8 ± 0.6	36.8 ± 0.5	36.6 ± 0.9	<0.001
Respiratory rate, bpm	18.0 ± 3.5	17.8 ± 3.3	20.0 ± 4.5	<0.001
MAP, mmHg	75.9 ± 9.2	76.2 ± 8.8	73.3 ± 11.5	<0.001
Heart rate, bpm	82.7 ± 13.8	82.2 ± 13.3	87.0 ± 17.1	<0.001
SpO_2_, %	91.8 ± 7.3	92.2 ± 6.4	87.7 ± 12.6	<0.001
**Comorbidities**				
CHF, %	4,067 (30.6)	3,479 (29.0)	588 (46.2)	<0.001
CHF–acute, %	2,283 (17.2)	1,893 (15.8)	390 (30.6)	<0.001
CHF–systolic, %	2,546 (19.2)	2,108 (17.6)	438 (34.4)	<0.001
HTN, %	7,059 (53.2)	6,590 (54.9)	469 (36.8)	<0.001
CAD, %	7,970 (60.0)	7,449 (62.0)	521 (40.9)	<0.001
AMI, %	1,650 (12.4)	1,408 (11.7)	242 (19.0)	<0.001
VA, %	1,185 (8.9)	965 (8.0)	220 (17.3)	<0.001
Diabetes, %	3,968 (29.9)	3,914 (30.1)	354 (27.8)	0.089
COPD, %	164 (1.2)	128 (1.1)	36 (2.8)	<0.001
Pneumonia, %	1,516 (11.4)	1,212 (10.1)	304 (23.9)	<0.001
Sepsis, %	321 (2.4)	150 (1.2)	171 (13.4)	<0.001
AKI, %	1,597 (12.0)	1,235 (10.3)	362 (28.4)	<0.001
CKD, %	869 (6.5)	740 (6.2)	129 (10.1)	<0.001
Liver disease, %	381 (2.9)	308 (2.6)	73 (5.7)	<0.001
Hepatitis, %	298 (2.2)	259 (2.2)	39 (3.1)	0.038
MELD score	15.8 ± 9.0	13.5 ± 7.0	24.2 ± 10.4	<0.001
Stroke, %	398 (3.0)	296 (2.5)	102 (8.0)	<0.001
Cancer, %	1,898 (14.3)	1,663 (13.9)	235 (18.5)	<0.001
**Laboratory tests**				
WBC, × 10^∧^9/L	12.2 ± 5.8	12.0 ± 5.4	14.2 ± 8.3	<0.001
Platelet, × 10^∧^9/L	198.8 ± 86.9	199.7 ± 84.6	218.6 ± 104.0	<0.001
Hemoglobin, g/dL	10.9 ± 2.5	10.9 ± 2.5	11.4 ± 3.1	<0.001
Sodium, mmol/L	137.4 ± 3.8	137.4 ± 3.6	137.7 ± 5.6	0.096
Potassium, mmol/L	4.4 ± 1.0	4.4 ± 1.0	4.6 ± 1.5	<0.001
Chlorine, mmol/L	105.7 ± 5.3	105.9 ± 5.1	104.0 ± 6.9	<0.001
Glucose, mmol/L	7.6 ± 2.2	7.5 ± 1.9	8.8 ± 3.6	<0.001
Creatinine, mg/dL	1.2 ± 1.1	1.1 ± 1.1	1.7 ± 1.3	<0.001
Urea nitrogen, mg/dL	22.2 ± 16.3	20.9 ± 14.7	34.8 ± 23.5	<0.001
Urine output (1st 24 h), mL/kg/h	1.08 ± 0.72	1.10 ± 0.69	0.91 ± 0.95	<0.001
<0.5, %	2,566 (19.3)	1,996 (16.6)	570 (44.8)	
0.5–1.0, %	4,764 (35.9)	4,446 (37.0)	318 (25.0)	
1.0-1.5, %	3,114 (23.5)	2,972 (24.8)	142 (11.2)	
1.5-2.0, %	1,485 (11.2)	1,416 (11.8)	69 (5.4)	
≥2.0, %	1,350 (10.2)	1,176 (9.8)	174 (13.7)	
Diuretic, %	9,358 (70.5)	8,640 (72.0)	718 (56.4)	<0.001

*CCU, coronary care unit; CSRU, cardiac surgery recovery unit; SAPS, simplified acute physiology score; SOFA, sequential organ failure assessment; MAP, mean aortic pressure; SpO2, oxyhemoglobin saturation; CHF, congestive heart failure; HTN, hypertension; CAD, coronary artery disease; AMI, acute myocardial infarction; VA, ventricular arrhythmia; COPD, chronic obstructive pulmonary disease; AKI, acute kidney injury; CKD, chronic kidney disease; MELD, model for end-stage liver disease; WBC, white blood cell*.

### Primary Outcomes

A multivariable logistic analysis was used to explore the association between UO and mortality. The low UO was a significant risk factor for 30-day mortality in CICU patients with odds ratio (OR) of 3.993 (95% CI: 3.447–4.625, *p* < 0.001). The UO in 1.0–1.5 ml/kg/h and 1.5–2.0 ml/kg/h were protective factors, with OR of 0.668 (95% CI: 0.545–0.819, *p* < 0.001) and 0.681 (95% CI: 0.522–0.890, *p* = 0.005). However, the association between UO and mortality was not negative linear, UO ≥ 2.0 ml/kg/h was also a remarkable risk factor for 30-day mortality [OR = 2.069 (95% CI: 1.701–2.516), *p* < 0.001]. In total, twenty-four variables were selected for adjusting the univariate logistic analysis by using a stepwise backward elimination method (Table 2). And UO ≥ 2.0 ml/kg/h was an independent risk factor for 30-day mortality as shown in the adjusted LR model (OR = 1.771, 95% CI: 1.389–2.256, *p* < 0.001). To identify the multicollinearity between the 24 variables, VIF test was performed and showed that there was no significant multicollinearity, with all the VIFs of the variables were <4.0, the mean VIF was 1.19.

**Table 2:** Univariate and multivariate logistic regression analyses for 30-day mortality.

	**Univariate logistic analysis**		**Multivariate logistic analysis**	
**Variables**	**OR (95% CI)**	***P*** **-Value**	**OR (95% CI)**	***P*** **-Value**
Age	1.045 (1.040–1.050)	<0.001	1.034 (1.027–1.040)	<0.001
Male	0.667 (0.594–0.793)	<0.001	0.827 (0.716–0.957)	0.011
SAPS	1.173 (1.159–1.187)	<0.001	1.078 (1.060–1.097)	<0.001
SOFA score	1.277 (1.254–1.301)	<0.001	1.083 (1.053–1.114)	<0.001
Temperature	0.619 (0.562–0.683)	<0.001	0.730 (0.654–0.815)	<0.001
Respiratory rate	1.158 (1.142–1.175)	<0.001	1.075 (1.055–1.095)	<0.001
MAP	0.963 (0.956–0.969)	<0.001	0.998 (0.990–1.005)	0.535
Heart rate	1.025 (1.021–1.029)	<0.001	1.020 (1.015–1.025)	<0.001
SpO_2_	0.951 (0.946–0.957)	<0.001	0.980 (0.972–0.986)	<0.001
CHF	2.104 (1.872–2.365)	<0.001	1.124 (0.968–1.305)	0.124
AMI	1.767 (1.520–2.054)	<0.001	1.892 (1.567–2.285)	<0.001
VA	2.390 (2.038–2.804)	<0.001	2.387 (1.956–2.913)	<0.001
COPD	2.701 (1.857–3.927)	<0.001	1.527 (0.962–2.426)	0.073
Sepsis	12.265 (9.766–15.402)	<0.001	4.289 (3.214–5.723)	<0.001
AKI	3.466 (3.027–3.968)	<0.001	1.723 (1.448–2.051)	0.001
CKD	1.717 (1.410–2.090)	<0.001	1.055(0.827–1.346)	0.666
Liver disease	2.310 (1.778–3.002)	<0.001	2.110 (1.511–2.948)	<0.001
Stroke	3.446 (2.730–4.350)	<0.001	3.853 (2.899–5.121)	<0.001
Malignancy	1.408 (1.211–1.637)	<0.001	1.248 (1.040–1.498)	0.017
WBC	1.053 (1.044–1.062)	<0.001	1.020 (1.010–1.031)	<0.001
Sodium	1.021 (1.006–1.038)	0.008	1.014 (0.999–1.031)	0.074
Potassium	1.157 (1.103–1.213)	<0.001	0.850 (0.777–0.928)	<0.001
Creatine	1.317 (1.270–1.367)	<0.001	1.046 (0.995–1.099)	0.076
Diuretic	0.415 (0.365–0.472)	<0.001	0.508 (0.436–0.591)	<0.001
**Urine output, mL/kg/h**				
**Urine output (0.5–1.0)**	**Ref**.		**Ref**.	
<0.5	3.993 (3.447–4.625)	<0.001	2.023 (1.693–2.417)	<0.001
1.0–1.5	0.668 (0.545–0.819)	<0.001	0.775 (0.622–0.967)	0.024
1.5–2.0	0.681 (0.522–0.890)	0.005	0.777 (0.581–1.038)	0.087
≥2.0	2.069 (1.701–2.516)	<0.001	1.771 (1.389–2.256)	<0.001

*The abbreviations are as same in Table 1*.

**Figure 2:**
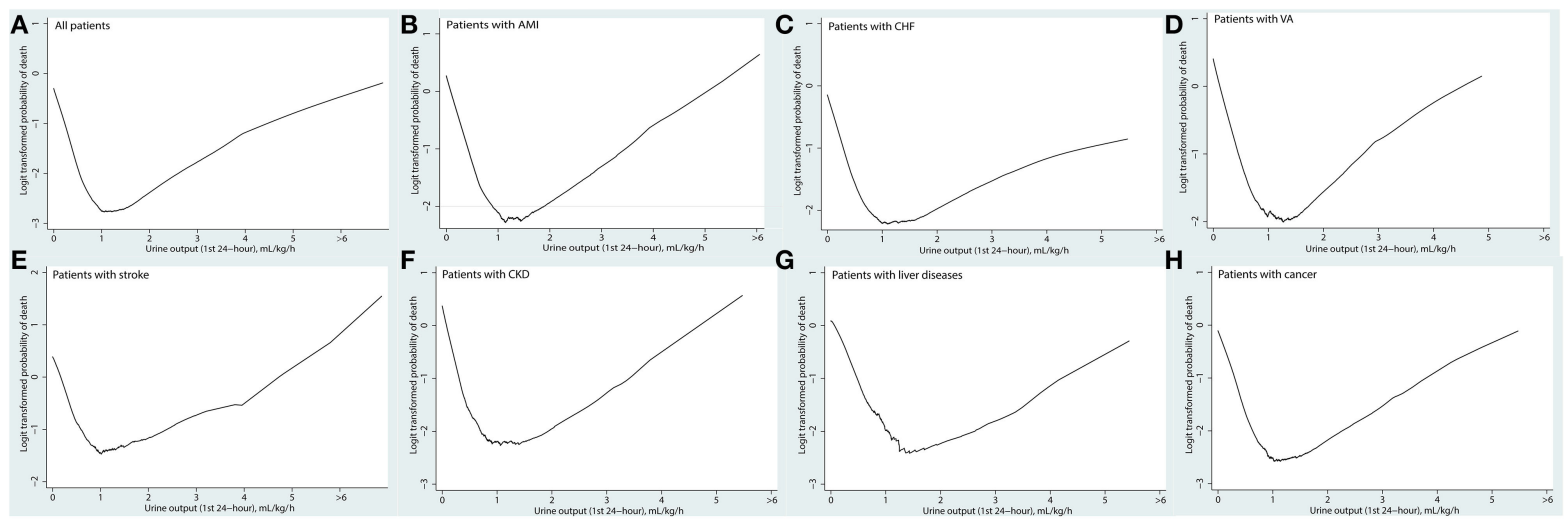
Association between urine output in the first 24 h and the 30-day mortality using the Lowess smoothing: **(A)** all patients; **(B)** patients with acute myocardial infarction; **(C)** patients with congestive heart failure; **(D)** patients with ventricular tachycardia or ventricular fibrillation; **(E)** patients with stroke including cerebral infarction and cerebral hemorrhage; **(F)** patients with chronic kidney disease; **(G)** patients with chronic liver diseases including chronic hepatitis and liver cirrhosis; **(H)** patients with cancer.

**Figure 3:**
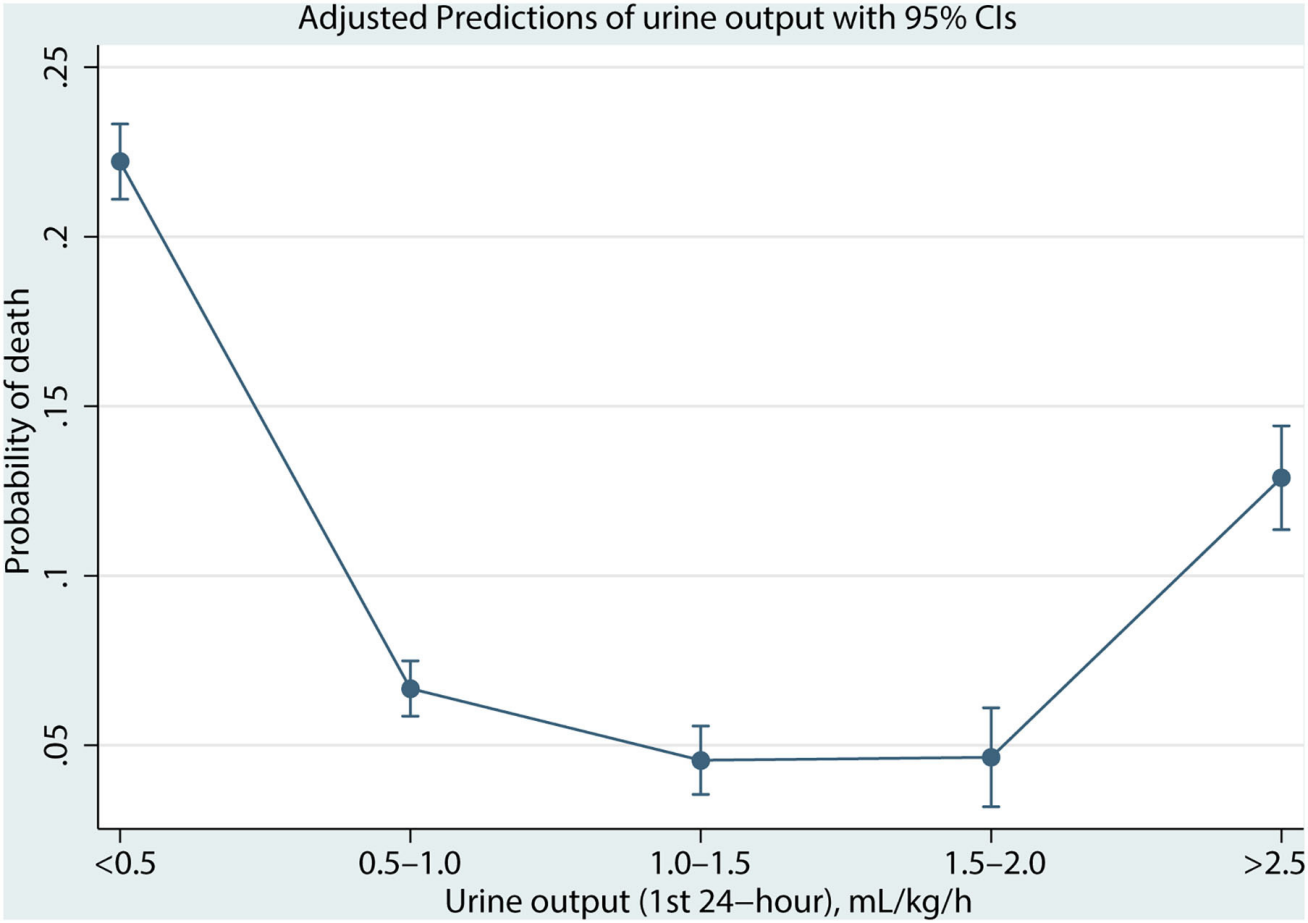
The predictive marginal effect of urine output in different ranges.

As shown in Figure 2, the association between UO and mortality is non-linear using the Lowess Smoothing technique. A U-curve relationship between UO and 30-day mortality was depicted in Figure 2A. We also explored the crude relationship between UO and the 30-day mortality in seven subgroups using the Lowess Smoothing technique. The U-shape association between UO and mortality was found in the seven subgroups as well (Figures 2B–H). The predictive marginal effects of the UO on 30-day mortality were shown in Figure 3. As the increase of UO in the first 24-h of ICU admission, the death probability of 30-day was not linear decline. Instead, there was a U-curve relationship between UO and mortality, when UO ≥ 2.0 ml/kg/h, the death probability would be significantly increased, which was consistent with the aforementioned findings.

### Secondary Outcomes

As shown in Table 3, low UO was strongly associated with 90-day mortality, ICU mortality and hospital mortality, with OR of 3.558 (95% CI: 3.116–4.064, *p* < 0.001), 4.072 (95% CI: 3.396–4.884, *p* < 0.001), and 3.950 (95% CI: 3.357–4.468, *p* < 0.001), respectively. Very high UO was also the important risk factor for 90-day mortality, ICU mortality and hospital mortality, with OR of 1.814 (95% CI: 1.517–2.169, *p* < 0.001), 1.998 (95% CI: 1.561–2.558, *p* < 0.001), and 1.561 (95% CI: 1.234–1.975, *p* < 0.001), respectively.

**Table 3:** Secondary outcomes.

**Variables**	**Non-adjusted**		**Model 1**		**Model 2**	
	**OR**	***P*** **-Value**	**OR**	***P*** **-Value**	**OR**	***P*** **-Value**
**90-day mortality**						
**0.5–1.0**	**Ref**.		**Ref**.		**Ref**.	
<0.5	3.558 (3.116**–**4.064)	<0.001	3.485 (3.044**–**3.989)	<0.001	1.893 (1.613**–**2.222)	<0.001
1.0–1.5	0.718 (0.604**–**0.853)	<0.001	0.738 (0.620**–**0.878)	0.001	0.833 (0.690**–**1.005)	0.057
1.5–2.0	0.724 (0.577**–**0.908)	0.005	0.763 (0.607**–**0.960)	0.021	0.828 (0.647**–**1.060)	0.133
≥2.0	1.814 (1.517**–**2.169)	<0.001	2.052 (1.709**–**2.465)	<0.001	1.619 (1.301**–**2.015)	<0.001
**ICU mortality**						
**0.5–1.0**	**Ref**.		**Ref**.		**Ref**.	
<0.5	4.072 (3.396**–**4.884)	<0.001	3.914 (3.260**–**4.700)	<0.001	1.545 (1.231**–**1.939)	<0.001
1.0–1.5	0.656 (0.505**–**0.852)	0.002	0.670 (0.515**–**0.871)	0.003	0.751 (0.563**–**1.002)	0.052
1.5–2.0	0.561 (0.388**–**0.812)	0.002	0.582 (0.401**–**0.843)	0.004	0.622 (0.417**–**0.926)	0.019
≥2.0	1.998 (1.561**–**2.558)	<0.001	2.150 (1.675**–**2.761)	<0.001	1.109 (0.790**–**1.557)	0.551
**Hospital mortality**						
**0.5–1.0**	**Ref**.		**Ref**.		**Ref**.	
<0.5	3.950 (3.357**–**4.648)	<0.001	3.812 (3.234**–**4.494)	<0.001	1.698 (1.386**–**2.080)	<0.001
1.0–1.5	0.633 (0.501**–**0.799)	<0.001	0.648 (0.513**–**0.819)	<0.001	0.737 (0.570**–**0.953)	0.020
1.5–2.0	0.632 (0.464**–**0.861)	0.004	0.660 (0.484**–**0.901)	0.009	0.716 (0.510**–**1.004)	0.053
≥2.0	1.561 (1.234**–**1.975)	<0.001	1.707 (1.345**–**2.167)	<0.001	0.899 (0.650**–**1.244)	0.522
**Ventilation**						
**0.5–1.0**	**Ref**.		**Ref**.		**Ref**.	
<0.5	1.258 (1.141**–**1.386)	<0.001	1.230 (1.116**–**1.357)	<0.001	1.720 (1.458**–**2.029)	<0.001
1.0–1.5	0.943 (0.859**–**1.034)	0.212	0.940 (0.856**–**1.032)	0.191	0.903 (0.775**–**1.052)	0.192
1.5–2.0	0.982 (0.872**–**1.107)	0.772	0.974 (0.863**–**1.098)	0.665	1.092 (0.894**–**1.333)	0.388
≥2.0	1.360 (1.204**–**1.536)	<0.001	1.331 (1.177**–**1.505)	<0.001	1.647 (1.333**–**2.035)	<0.001
**Vasopressor**						
**0.5–1.0**	**Ref**.		**Ref**.		**Ref**.	
<0.5	1.096 (0.995**–**1.207)	0.063	1.087 (0.986**–**1.198)	0.093	1.358 (1.194**–**1.545)	<0.001
1.0–1.5	0.995 (0.908**–**1.090)	0.917	0.979 (0.893**–**1.073)	0.649	0.943 (0.842**–**1.056)	0.331
1.5–2.0	0.925 (0.821**–**1.041)	0.193	0.889 (0.790**–**1.002)	0.054	0.825 (0.712**–**0.956)	0.011
≥2.0	1.178 (1.043**–**1.330)	0.008	1.093 (0.967**–**1.236)	0.156	0.971 (0.829**–**1.138)	0.719

*Model 1: age, gender. Model 2: age, male, Simplified Acute Physiology Score, Sequential Organ Failure Assessment score, temperature, respiratory rate, mean aortic pressure, heart rate, oxyhemoglobin saturation, congestive heart failure, ventricular arrhythmia, acute myocardial infarction, chronic obstructive pulmonary disease, sepsis, acute kidney injury, chronic kidney disease, liver diseases, stroke, malignancy, white blood cell, sodium, potassium, creatinine, diuretic*.

In model 1, which was adjusted by age and gender, UO ≥ 2.0 ml/kg/h was an independent risk factor, with OR of 2.052 (95% CI: 1.709–2.465, *p* < 0.001), 2.150 (95% CI: 1.675–2.761, *p* < 0.001), 1.707 (95% CI: 1.345–2.167, *p* < 0.001), and 1.331 (95% CI: 1.177–1.505, *p* < 0.001) for 90-day mortality, ICU mortality, hospital mortality, and use of MV, respectively. In model 2, which was adjusted by age, gender, SAPS, SOFA score, temperature, RR, MAP, HR, SpO2, CHF, VA, AMI, COPD, sepsis, AKI, CKD, liver diseases, stroke, cancer, WBC, sodium, potassium, creatine, and use of diuretics, UO ≥ 2.0 ml/kg/h was associated with 90-day mortality as well, with OR of 1.619 (95% CI: 1.301–2.015, *p* < 0.001). However, this association was insignificant for ICU and hospital mortality, with OR of 1.109 (95% CI: 0.790–1.557, *p* = 0.551) and 0.889 (95% CI: 0.650–1.244, *p* = 0.522). In order to further investigate the relationships between high UO and ICU and hospital mortality, we performed logistic regression analyses for patients with UO ≥ 2.5 ml/kg/h using the model 2. The results showed that UO ≥ 2.5 ml/kg/h was highly associated with ICU (OR = 1.730, 95% CI: 1.164–2.573, *p* = 0.007) and hospital mortality (OR = 1.622, 95% CI: 1.121–2.347, *p* = 0.010). Other secondary outcomes were exhibited in Table 3.

### Subgroup Analysis

**Figure 4:**
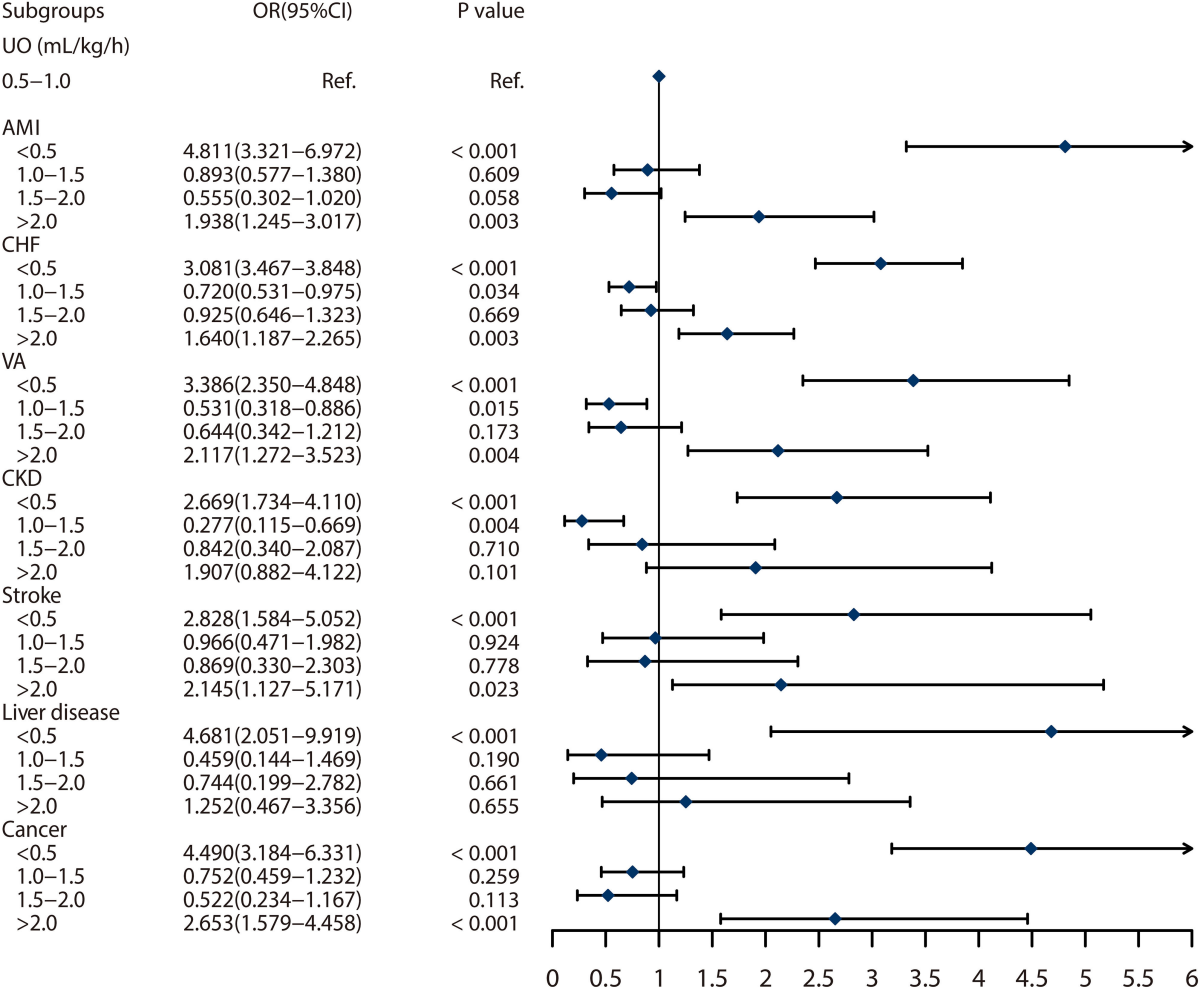
Subgroup analyses on association between urine output and 30 days mortality based on the different comorbidities. The abbreviations are as same in Table 1.

Seven subgroups were included in the present study. We found that UO ≥ 2.0 ml/kg/h was associated with higher 30-day mortality compared with the reference, with OR of 1.938 (95% CI: 1.245–3.017, *p* = 0.003), 1.640 (95% CI: 1.187–2.265, *p* = 0.003), 2.117 (95% CI: 1.272–3.523, *p* = 0.004), 2.145 (95% CI: 1.127–5.171, *p* = 0.023), and 2.653 (95% CI: 1.579–4.458, *p* < 0.001), in patients with AMI, CHF, VA, stroke or cancer, respectively. However, the effect was not shown in patients with CKD and liver disease. In addition, low UO was also found a risk factor for 30-day mortality in all the subgroups. In summary, abnormal UO was independently associated with the 30-day mortality in patients with AMI, CHF, VA, CKD, COPD, chronic liver disease, and cancer (Figure 4).

## Discussion

The 30-day mortality in critical ill patients in this study was about 10%, which was consistent with the previous studies (14–16). In this study, we investigated the relationship between UO and short-term mortality in the CICU patients. The main findings are as follows: (1) Low UO (<0.5 ml/kg/h) and very high UO (≥2.0 ml/kg/h) are both independently associated with an increased risk of 30-day mortality, and there is a U-shape relationship between the mortality and UO instead of a linear correlation reported by the previous study (10); (2) Abnormal UO is also highly associated with 90-day mortality, ICU mortality, hospital mortality, use of MV and vasopressor agents; (3) Low or very high UO may significantly increase the mortality in patients with AMI, CHF, VA, CKD, stroke, chronic liver disease and cancer, which are common diseases in the CICU.

Urine output is an efficient marker reflecting the renal function (17). Oliguria has been demonstrated a powerful predictive factor for AKI. Some studies found that UO could provide an earlier indication of the renal dysfunction than SCr, and AKI would be diagnosed at least 1 day earlier when UO was considered (9, 18). For the patients hospitalized in the CICU, the change of SCr is lagging to reflect renal function, contributing to a delayed diagnosis of AKI (19). As an inexpensive and widely available method, UO also serves as a strong prognostic indicator and oliguria is strongly associated with an increased mortality (20–22). In addition, isolated oliguria without AKI is also highly associated with the adverse outcomes (9). However, the precise mechanisms of the relationship between decreased UO and poor prognosis are still unclear. One reason could be that oliguria is an important predictive factor of the renal dysfunction, which subsequently increases the mortality. It is note-worthy that, in some cases, decreased UO are not followed by the renal disorder, while the transient oliguria may be induced by hypovolemia and can be reversed with adequate resuscitation. In brief, patients with oliguria would suffer from adverse outcomes which was consistent with our findings.

Usually, clinicians pay more attention to oliguria than polyuria in the critical care (23). In fact, not only decreased UO could result in a poor prognosis, increased UO is highly associated with the adverse outcomes as well. To our knowledge, there are limited studies focusing on the relationship between polyuria and mortality in patients with critical cardiovascular illness. We found that the mean of the first 24-h UO ≥ 2.0 ml/kg/h was significantly associated with increased mortality. Although it is widely acknowledged that patients with oliguria is more often than polyuria in CICU. And there are some common reasons accounting for polyuria in CICU, including heavy FI and use of diuretics. The precise mechanisms by which polyuria increases mortality, however, are still not fully understood. Some pathophysiology processes may help explaining this relationship. On the one hand, polyuria, especially caused by overdose of diuretics, could lead to hypovolemia, hypotension and even shock, which are associated with high mortality (24–26). Moreover, diuretics are commonly used in CICU to reduce the cardiac volume load (27, 28). In this study, about 70% of the participants used diuretics. On the other hand, diuretics could interfere electrolyte balance, including anomalies of potassium and sodium (29, 30), which could result in the poor outcomes. In this study, we found that hypernatremia and hyperkalemia may be the risk factors for 30-day mortality in the univariate logistic analysis, with OR of 1.021 (95% CI: 1.006–1.038, *p* = 0.008) and 1.157 (95% CI: 1.103–1.213, *p* < 0.001), respectively. However, the relationships were not found in the multivariate logistic analysis. In this study, FI was not included for analysis. Nevertheless, heavy FI may induce hemodilution which could influence the true concentration of electrolyte including potassium and sodium. Moreover, the drugs used to correct electrolyte disturbances were not included in the logistic regression. Therefore, the relationships between electrolyte and mortality in this study should be interpreted with caution.

Aggressive fluid resuscitation plays an important role in maintaining adequate organ perfusion in the immediate postoperative period for the cardiac surgical patients (31). Moreover, UO was significantly affected by FI. And Shen et al. found that the association between UO and mortality was mediated by the UO/FI status, and was only significant in the subgroup with UO/FI ratios of ≤ 0.5 (32). In this study, however, the logistic regression models were not adjusted by FI, which may have influence on the results.

In addition, many patients in CICU are vulnerable to respiratory failure and hemodynamic instability, requiring MV and vasopressor agents to stabilize condition. And those on pressor or MV for an extended period of time tend to have a poor prognosis (33, 34). In this study, we found that abnormal UO was significantly associated with the use of MV and vasopressor agents. Accordingly, we acclaim that both the decreased and increased UO could be the strong risk factors for adverse outcomes in patients admitted to CICU.

Patients hospitalized in CICU are commonly accompanied by the multi-organ disorders and a variety of comorbidities including stroke, liver disorders, and cancer, which may affect the study results. Therefore, we performed seven subgroup analyses to adjust our findings. And we reached the same conclusion that both decreased and increased UO were associated with poor outcomes in patients with AMI, CHF, VA, CKD, stroke, chronic liver disease, and cancer.

## Limitations

Although this was a large sample cohort study that reached a new finding, this study also had some limitations. First, this was a retrospective, single center study, and a prospective clinical trial was needed. Second, there were some missing and extreme data in the MIMIC-III database, some variables with too much missing data, including cardiac troponin and B-type natriuretic peptide, were excluded. And some missing data were filled using specific methods, which may have some influence on the results. Finally, FI in the first 24-h was not included in this study. Although UO was a valid and easily obtained factor reflecting the circulatory situation, it was also influenced by FI, which may provide bias.

## Conclusion

Low and very high UO both are independently associated with the short-term mortality and the use of MV and vasopressor agents in patients admitted for cardiovascular diseases. The relationship between UO and mortality was *U*-shape rather than linear. Prospective cohort studies are needed to support the findings.

## Data Availability Statement

Publicly available datasets were analyzed in this study. This data can be found at: https://mimic.mit.edu/.

## Ethics Statement

Ethical review and approval was not required for the study on human participants in accordance with the local legislation and institutional requirements. Written informed consent for participation was not required for this study in accordance with the national legislation and the institutional requirements.

## Author Contributions

This study was designed by LL. ZHZ, YLX, ZH, SYL, and BT were responsible for data collation and statistical analysis. LL was responsible for data extraction and wrote the first draft. YY reviewed and checked the manuscript. All the authors read and approved the final manuscript.

## Funding

This study was supported by the National Natural Science Foundation of China (Grant No. 81970285).

## Conflict of Interest

The authors declare that the research was conducted in the absence of any commercial or financial relationships that could be construed as a potential conflict of interest.

## Publisher's Note

All claims expressed in this article are solely those of the authors and do not necessarily represent those of their affiliated organizations, or those of the publisher, the editors and the reviewers. Any product that may be evaluated in this article, or claim that may be made by its manufacturer, is not guaranteed or endorsed by the publisher.
